# Nanolaminate-based design for UV laser mirror coatings

**DOI:** 10.1038/s41377-020-0257-4

**Published:** 2020-02-11

**Authors:** Meiping Zhu, Nuo Xu, Behshad Roshanzadeh, S. T. P. Boyd, Wolfgang Rudolph, Yingjie Chai, Jianda Shao

**Affiliations:** 1 Laboratory of Thin Film Optics, Shanghai Institute of Optics and Fine Mechanics, Chinese Academy of Sciences, Shanghai, 201800 China; 2 Center of Materials Science and Optoelectronics Engineering, University of Chinese Academy of Sciences, Beijing, 100049 China; 3 Hangzhou Institute for Advanced Study, University of Chinese Academy of Sciences, Hangzhou, 310024 China; 4 Key Laboratory of Materials for High Power Laser, Shanghai Institute of Optics and Fine Mechanics, Chinese Academy of Sciences, Shanghai, 201800 China; 5 Department of Physics and Astronomy, University of New Mexico, Albuquerque, NM 87131 USA; 6 CREOL, The College of Optics and Photonics, University of Central Florida, Orlando, FL 32816 USA

**Keywords:** Solid-state lasers, Optical materials and structures

## Abstract

With ever-increasing laser power, the requirements for ultraviolet (UV) coatings increase continuously. The fundamental challenge for UV laser-resistant mirror coatings is to simultaneously exhibit a high reflectivity with a large bandwidth and high laser resistance. These characteristics are traditionally achieved by the deposition of laser-resistant layers on highly reflective layers. We propose a “reflectivity and laser resistance in one” design by using tunable nanolaminate layers that serve as an effective layer with a high refractive index and a large optical bandgap. An Al_2_O_3_–HfO_2_ nanolaminate-based mirror coating for UV laser applications is experimentally demonstrated using e-beam deposition. The bandwidth, over which the reflectance is >99.5%, is more than twice that of a traditional mirror with a comparable overall thickness. The laser-induced damage threshold is increased by a factor of ~1.3 for 7.6 ns pulses at a wavelength of 355 nm. This tunable, nanolaminate-based new design strategy paves the way toward a new generation of UV coatings for high-power laser applications.

The demand for laser-resistant mirror coatings is increasing in inertial confinement fusion (ICF)^1^, extreme light infrastructure^2^ and other laser applications^3–6^. An ideal ultraviolet (UV) laser mirror (UVLM) coating requires a high reflectivity with a large bandwidth and a high laser-induced damage threshold (LIDT). Unfortunately, these requirements are difficult to satisfy simultaneously, because, for example, a high reflectivity requires materials with a high refractive index (*n*), while higher *n* materials tend to have a smaller optical bandgap and therefore a lower LIDT. Traditionally, compromises are made for these seemingly contradictory requirements^7–10^. We propose to use nanolaminate coatings for UVLMs. Nanolaminate materials^11–16^ have properties that make them attractive for many applications^15,17–20^. Our nanolaminate-based UVLM coatings are deposited using e-beam evaporation, a technique that is particularly favorable for large laser optics^10,21–24^. This novel concept results in improved performance parameters and paves the way toward a new generation of UV coatings for high-power laser applications.

In the traditional “reflectivity bottom and LIDT top” combination design (TCD coating) strategy, alternating high-*n* and low-*n* layers, such as HfO_2_ and SiO_2_, are deposited on the substrate to obtain a high reflectivity, as illustrated in Fig. 1a (the high-*n* layer is denoted as *C*_*HN*_). Subsequently, pairs of high-*n* layers (with a relatively larger optical bandgap than *C*_*HN*_, denoted as layer C_*LB*_) and low-*n* layers, such as Al_2_O_3_ and SiO_2_, or LaF_3_ and AlF_3_, are deposited to achieve a high LIDT^8,9^. Our new “reflectivity and laser resistance in one” strategy uses nanolaminate layers with co-evaporated interfaces, which are produced by alternating C_*LB*_ layers and C_*HN*_ layers, as shown in Fig. 1b. The C_*LB*_-C_*HN*_ nanolaminate layers can be considered high-*n* layers, with a tunable refractive index and optical bandgap. As we will show, this arrangement allows for mirror designs with the advantageous combination of a high reflectivity and a high LIDT.

**Fig. 1:**
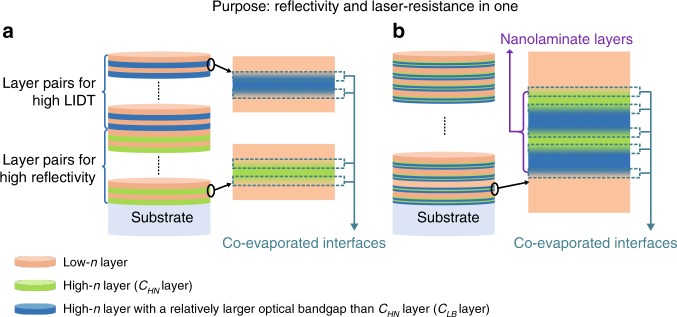
Schematic diagram of the stacks of optical coatings for HR mirrors with a high LIDT. **a** Traditional design using pairs of high- and low-index materials. **b** Proposed strategy using nanolaminate layers and low-index materials

The high-*n* materials HfO_2_ and Al_2_O_3_ in combination with the low-*n* material SiO_2_ are widely used for multilayer coating stacks in the UV region^25^. The microstructure and optical properties of an e-beam-evaporated Al_2_O_3_–HfO_2_ nanolaminate coating consisting of 18 Al_2_O_3_–HfO_2_ pairs have been compared with those of HfO_2_ and Al_2_O_3_ single-layer coatings. In this study, ~4-nm co-evaporated interfaces are introduced for each alternating interface between two different materials, as shown in Fig. 1. The elemental percentages of an Al_2_O_3_–HfO_2_ pair in the nanolaminate vs. depth are measured by XPS and are shown in Fig. 2a. Due to diffusion, both the Al_2_O_3_ and HfO_2_ contents are observed at each depth. The Al_2_O_3_ to HfO_2_ integrated content ratio in an Al_2_O_3_–HfO_2_ pair is calculated to be ~1.7:1. A sharp diffraction peak is not observed in the X-ray diffraction (XRD) spectra of the Al_2_O_3_–HfO_2_ nanolaminate and Al_2_O_3_ single-layer coatings, while multiple sharp diffraction peaks indicative of crystallinity are obtained from the HfO_2_ single-layer coating (Fig. 2b). The transmittance spectra of the substrate and coatings are measured to determine the refractive indices (Fig. 2c). The optical bandgaps of the Al_2_O_3_ and HfO_2_ single-layer coatings and Al_2_O_3_–HfO_2_ nanolaminate coatings are estimated using the Tauc equation^26^ to be 6.25 eV, 5.44 eV, and 5.65 eV, respectively. The Al_2_O_3_–HfO_2_ nanolaminate coating acts as an equivalent single-layer coating with a higher refractive index than Al_2_O_3_ and a larger optical bandgap than HfO_2_. The (average) refractive index and optical bandgap can be tuned by adjusting the thickness ratio of the two materials in the nanolaminate layers while keeping the total optical thickness constant. This allows one to develop UVLM coatings with a high reflectivity and a high LIDT.

**Fig. 2:**
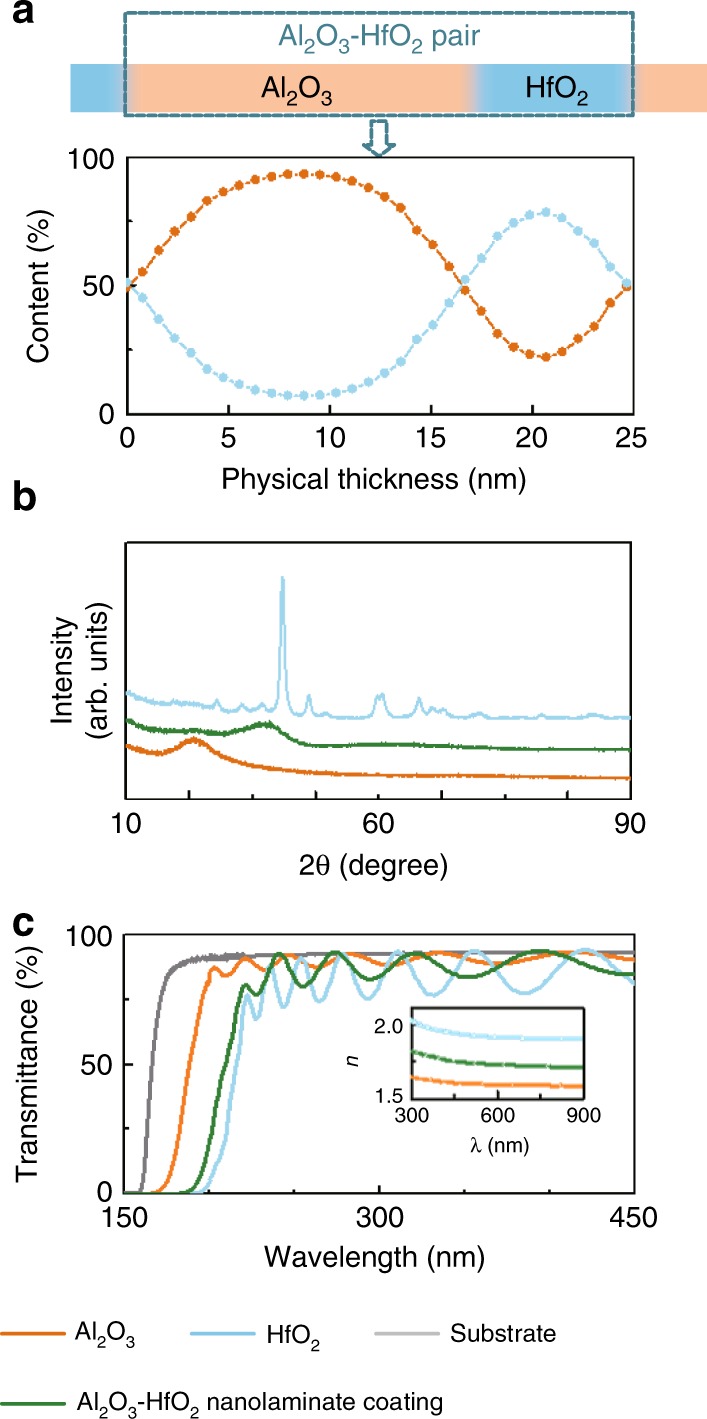
The microstructure and optical properties of Al_2_O_3_–HfO_2_ nanolaminate, Al_2_O_3_ and HfO_2_ single layer coatings. **a** Al_2_O_3_ and HfO_2_ content as a function of physical thickness in a nanolaminate coating. **b** XRD spectra and **c** transmittance of an Al_2_O_3_ single layer, an HfO_2_ single layer and an Al_2_O_3_–HfO_2_ nanolaminate coating with physical thicknesses of 513.8 nm, 540.7 nm, and 444.1 nm, respectively. The inset shows the corresponding refractive indices

Using the refractive index and optical bandgap tunability, a nanolaminate-based multilayer coating (NLD coating) consisting of Al_2_O_3_–HfO_2_ nanolaminate layers with an equivalent *n* of 1.714 at 355 nm is designed. The coating structure is as follows: substrate/4L(0.335A0.165H0.335A0.165HL)^20^A8.15L/air. Here, A, H, and L represent Al_2_O_3_, HfO_2_, and SiO_2_ layers with a quarter-wavelength optical thickness (QWOT), respectively. The number represents the optical thickness in units of the QWOT of the respective material. Thus, 0.335A0.165H0.335A0.165H represents a nanolaminate layer with four sublayers (Supplementary Fig. S2). For a given total optical thickness, a nanolaminate layer with a larger number of sublayers has a higher optical bandgap^16^. Here, four sublayers are used as a compromise between a large bandgap and the overall layer quality. Note that the co-evaporated interfaces have a thickness of ~4 nm. To achieve a high reflectivity at 355 nm for an angle of incidence of 45°, the reference wavelength *λ*_0_ is 395 nm. The refractive indices of A, H, and L at 395 nm are 1.616, 1.956, and 1.481, respectively. According to 2 × (*n*_A_*d*_0.335A_ + *n*_H_*d*_0.165H_) = *n*_L_*d*_L_ = *n*_A_*d*_A_ = λ_0_/4, the physical thicknesses are *d*_4L_ = 266.80 nm, *d*_0.335A_ = 20.47 nm, *d*_0.165H_ = 8.33 nm, *d*_L_ = 66.70 nm, *d*_A_ = 61.11 nm, and *d*_8.15L_ = 543.61 nm. The designed Al_2_O_3_ to HfO_2_ integrated content ratio in the nanolaminate layers is 2.46:1. A thick SiO_2_ overcoat layer (8.15L) is used as a protective layer, and the resulting electric (E)-field intensity at the coating-air interface is close to 0.

For a comparison, the aforementioned NLD coating stack and a TCD coating with a substrate/4L(HL)^5^(AL)^15^A8.15L/air structure are prepared. Obvious sharp diffraction peaks are not observed in the XRD spectra of either the NLD and TCD coatings. Figure 3a, b shows images from high-resolution transmission electron microscopy (TEM) of both coatings, and the lattice is consistent with the selected-area electron diffraction result (see Supplementary Fig. S4). The elemental percentages vs. depth are shown in Fig. 3c. The Al_2_O_3_ to HfO_2_ integrated content ratio in the nanolaminate layers is ~2.2:1, slightly lower than the designed value. Al_2_O_3_ is also observed at the interface between SiO_2_ and HfO_2_ in the nanolaminate layers, which is attributed to diffusion and/or preferential sputtering effects. The reflectance spectra of the two coatings are compared in Fig. 3d. The high-reflectivity (*R*s ≥ 99.5%) bandwidth of the NLD coating is 38 nm, more than twice that of the TCD coating (17 nm). In addition, the NLD average transmission is higher in the VIS–NIR region and exhibits smaller ripple amplitudes (Fig. 3e). This result indicates that properly designed NLD structures also have potential as harmonic separators, which require a high reflectivity at one wavelength and a high transmittance at other wavelengths.

**Fig. 3:**
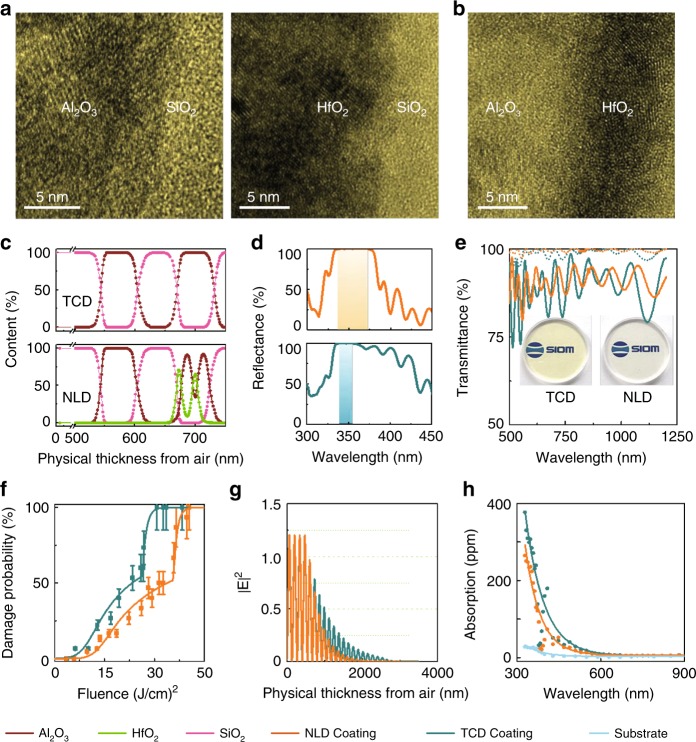
The microstructure and optical properties of the TCD and NLD coatings. High-resolution transmission electron microscopy of the (**a**) TCD and (**b**) NLD coatings. **c** SiO_2_, Al_2_O_3_, and HfO_2_ content vs. depth, **d** reflectance, and **e** transmittance spectra (incident angle of 45°, *s*-polarized light indicated by solid lines, and *p*-polarized light indicated by dotted lines), **f** single-pulse damage probability as a function of the input fluence, **g** E-field distribution, and **h** optical absorption of the TCD and NLD coatings vs. wavelength

The laser damage probabilities as a function of fluence of the two coating structures are compared in Fig. 3f. The damage probability curves show a shape that can, for example, be explained by the presence of two types of defects with different LIDTs. The defect parameters can be extracted using the model developed by Krol et al.^27^. If we assume the existence of two defects with different values of the LIDT *T*_*i*_ and area density *D*_*i*_ (integrated over the thickness), we obtain the results shown in Table 1. Overall, the NLD coating shows improved damage resistance compared with the damage resistance of the TCD coating stack.

**Table 1:** Extracted defect parameters

	*D*_*1*_ (1/mm^2^)	*T*_*1*_ (J/cm^2^)	*ΔT*_*1*_ (J/cm^2^)	*D*_*2*_ (1/mm^2^)	*T*_*2*_ (J/cm^2^)	*ΔT*_*2*_ (J/cm^2^)
TCD	6.5	10.1	6.0	200.0	25.5	1.0
NLD	6.1	13.5	6.0	298.0	35.0	1.0

To investigate the damage mechanism, the stress, absorption, E-field distribution, and damage morphology of the coatings are studied. The stresses of the TCD and NLD coatings are determined to be −160.7 MPa and −171.1 MPa, respectively. The E-field intensity in the NLD coating decays more rapidly with depth than the TCD coating (Fig. 3g). The absorption of the coating stacks is measured using nano-Kelvin calorimetry^28^. At the wavelength of interest (355 nm), the absorption losses in the NLD coating stack are ~20% smaller than those in the TCD mirror (Fig. 3h). Consequently, the laser-induced temperature increase in the NLD coating will be lower.

Typical damage morphologies of the NLD and TCD structures are shown in Fig. 4 and suggest the existence of two types of defects. One morphological feature is related to nodules, which produce a pit for an illumination fluence just above the LIDT (Fig. 4i). At larger fluences, the top layers are ablated (Fig. 4j). As the laser fluence further increases, a second type of damage morphology can be observed. This morphology consists of many shallow pits (Fig. 4k), which evolve into larger areas of delaminated films for larger laser fluences (Fig. 4l). The defects that initiate damage features of this second type do not involve nodules. The damage morphology of the second type is observed for fluences of 25.9 J/cm^2^ in the TCD coatings (Fig. 4g), while only the first kind of morphology is observed in the NLD coatings for fluences at 32.7 J/cm^2^ (Fig. 4b). After laser irradiation with the same fluence above the LIDT, the damaged area in the NLD coating stack is smaller than that in the TCD coating stack.

**Fig. 4:**
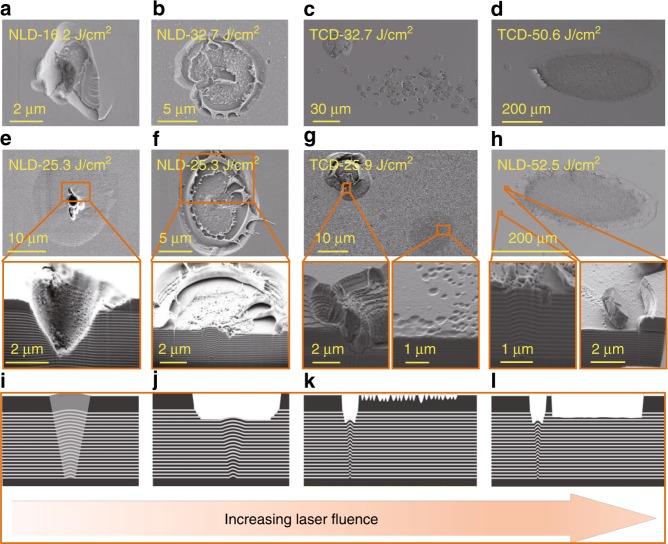
Damage morphologies of the TCD and NLD coatings. **a**–**h** Damage sites imaged by SEM and the depth profiles of the marked regions measured by FIB. **i**–**l** Schematic diagram of the simulated damage morphologies for increasing laser fluence

A finite-element method (FEM) simulation is used to illustrate the effect of nodular defects on the E-field intensity distribution. As an input for the simulation, the nodular defect size is estimated from the FIB cross-sections (Fig. 4). In both the TCD- and NLD-coating stacks, the nodular defects produce an E-field intensification (see Supplementary Fig. S5), especially in the top layers close to air. This result explains why laser damage originates from nodules, and a delamination of top layers occurs as the laser fluence increases. Compared with the TCD coating, the NLD coating exhibits a lower E-field intensification, faster E-field decay with depth and smaller absorption, which are consistent with the observed higher LIDT.

In summary, we have developed and experimentally demonstrated a new class of UVML coatings with an unprecedented combination of properties. The new structure replaces the high-*n* materials in traditional designs with nanolaminate layers. The proposed method enables UVML coatings with a larger high-reflectivity bandwidth, higher LIDT, and smaller transmission ripples in the VIS–NIR region than traditional designs with a comparable overall thickness. The e-beam-deposited nanolaminate materials can be used for large (meter-scale) UVML coatings. We believe that the described concept opens new avenues for improved UV coatings and can benefit many areas of laser technology that rely on high-quality optical coatings.

## Materials and methods

### Preparation of coatings

HfO_2_ and Al_2_O_3_ single-layer coatings, Al_2_O_3_–HfO_2_ nanolaminate coatings, and TCD and NLD multilayer coatings are deposited on fused silica substrates using e-beam evaporation. The coating chamber is heated to 473 K and evacuated to a base pressure of 9 × 10^–4^ Pa before deposition. The deposition rates for the HfO_2_, Al_2_O_3_, and SiO_2_ layers are 0.1 nm/s, 0.1 nm/s, and 0.2 nm/s, respectively. The oxygen pressure of HfO_2_ and Al_2_O_3_ is 1.3 × 10^–2^ Pa. Except for the 4L layer (3.0 × 10^–3^ Pa) in the TCD and NLD coatings, the oxygen pressure of the remaining SiO_2_ is 5.0 × 10^–3^ Pa. The co-evaporated interface is obtained by dual e-beam co-evaporation. The details of the co-evaporation system and process are shown in Supplementary Figs. S1 and S2.

### Characterization of the coatings

The transmittance spectra in the range of 150–220 nm and 220–1200 nm are measured by a VUV spectrometer (LZH ML 6500) and a UV–visible spectrometer (Perkin Elmer Lambda 1050), respectively. The reflectance spectra in the VIS–NIR region are calculated from the transmission data neglecting absorption. The refractive indices are fitted using commercial thin film software (Essential Macleod). The E-field intensity distributions are obtained from FEM simulations.

The elemental compositions along the depth are determined by X-ray photoelectron spectroscopy (XPS, Thermo Scientific K-Alpha) using a monochromatic Al Kα (1486.6 eV) X-ray source. The spectra are recorded after every 20 s of etching with 1 keV Ar^+^ ions.

The surface and cross-section morphologies of the laser-induced damaged sites are obtained by a focused ion beam scanning electron microscope (FIB-SEM, Carl Zeiss AURIGA CrossBeam).

The 1-on-1 LIDT is determined according to ISO 21254 using an *s*-polarized 3ω Nd: YAG laser with a Gaussian TEM_00_, single-longitudinal mode (355 nm). The pulse shape is Gaussian with a full width at half maximum of 7.6 ns. The test is performed at an angle of incidence of 45°. The effective beam size on the sample surface is ~0.18 mm^2^, and 15 sites are tested for each energy fluence. The sample surfaces before (substrate) and 15 days after the deposition are inspected by an interferometer (ZYGO Mark III-GPI) at 632.8 nm in an environment with a temperature of 23 ± 1.5 °C and a relative humidity of 45 ± 5%. The coating stress is obtained from Stoney’s formula. The absorption is measured by an optical nano-Kelvin calorimeter^28^ using a tunable light source with a spectral width of ~6 nm, a step size of 5 nm, and an optical illumination power between 30 and 40 µW.

## Supplementary information

Nanolaminate-based design for UV laser mirror coatings_supplemental materials
