# Supplementary material for: Nanolaminate-based design for UV laser mirror coatings
Source: Light Sci Appl. 2020 Feb 11;9:20. doi: 10.1038/s41377-020-0257-4 (PMC7012845; doi:10.1038/s41377-020-0257-4)
Supplement: Supplementary file 1 — Nanolaminate-based design for UV laser mirror coatings_supplemental materials [file 41377_2020_257_MOESM1_ESM.docx]

Supplementary Information

Nanolaminate-based design for UV laser mirror coatings

Meiping Zhu^1,2,3,4*^, Nuo Xu^1,2,4^, Behshad Roshanzadeh^5^, S. T. P. Boyd^5^,

Wolfgang Rudolph^5*^, Yingjie Chai^6^ and Jianda Shao^1,3,4*^

^1^Laboratory of Thin Film Optics, Shanghai Institute of Optics and Fine Mechanics, Chinese Academy of Sciences, Shanghai 201800, China

^2^Center of Materials Science and Optoelectronics Engineering, University of Chinese Academy of Sciences, Beijing 100049, China

^3^Hangzhou Institute for Advanced Study, University of Chinese Academy of Sciences, Hangzhou 310024, China

^4^Key Laboratory of Materials for High Power Laser, Shanghai Institute of Optics and Fine Mechanics, Chinese Academy of Sciences, Shanghai 201800, China

^5^Department of Physics and Astronomy, University of New Mexico, Albuquerque, NM 87131, USA

^6^CREOL, The College of Optics and Photonics, University of Central Florida, Orlando, FL 32816, USA

^*^Corresponding authors.

E-mail: [bree@siom.ac.cn](mailto:bree@siom.ac.cn) (M. Zhu); [wrudolph@unm.edu](mailto:wrudolph@unm.edu) (W. Rudolph); [jdshao@siom.ac.cn](mailto:jdshao@siom.ac.cn) (J. Shao).

The schematic diagram of the e-beam evaporation system used in this work is shown in Figure S1. Four e-beam guns (EBGs) and two separate quartz crystal monitors are equipped. The optical and physical thicknesses are monitored using an optical and crystal monitor system located in the top center of the system.


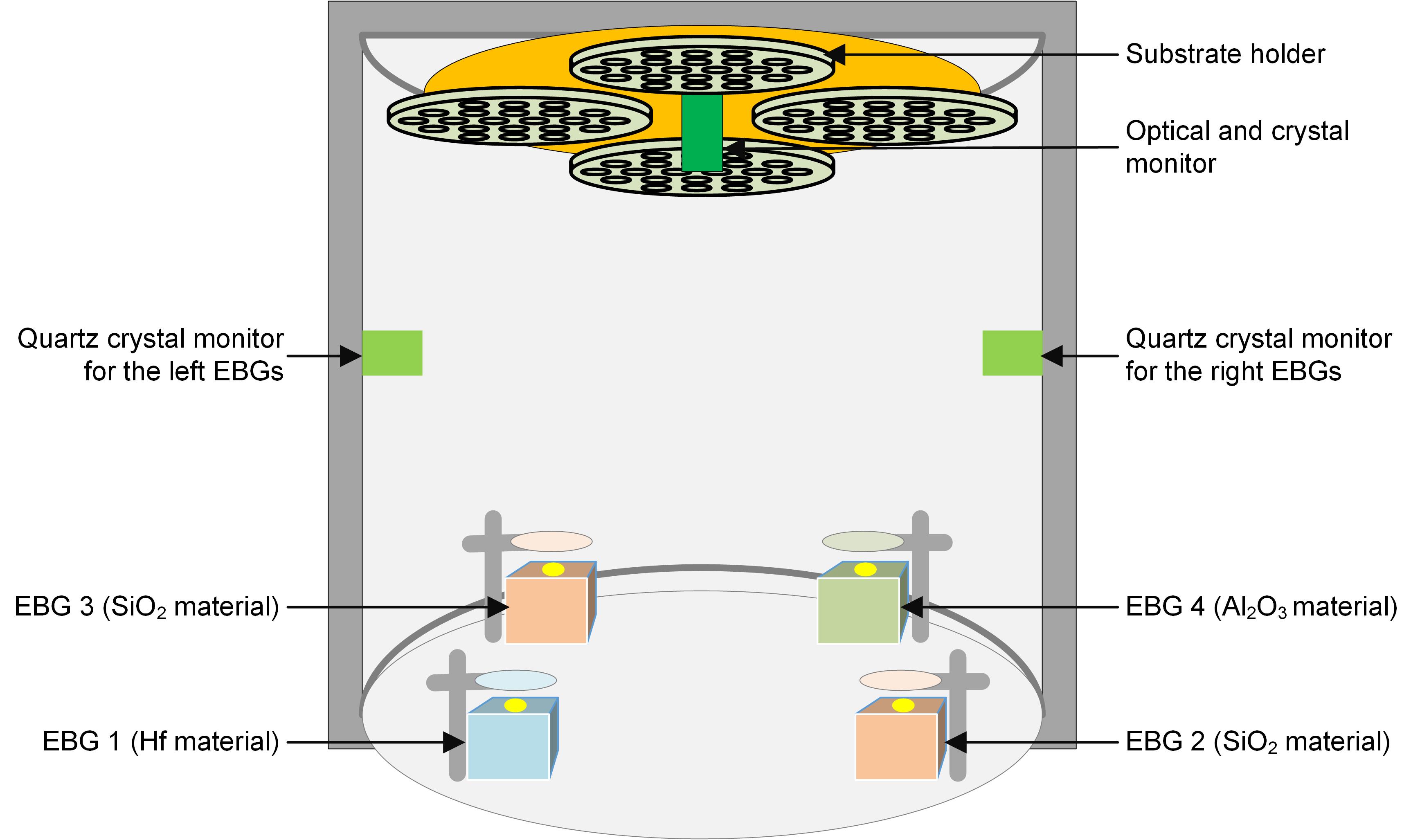


**Figure S1.** Schematic diagram of the e-beam evaporation system

The deposition rates of different materials and the EBGs used are indicated in Figure S2. The deposition rates of the materials are monitored by the quartz crystal monitor located at the corresponding side. For the deposition of the interfaces between different materials, one selected EBG on the left and the other selected EBG on the right work simultaneously. For example, EBG 3 and EBG 4 evaporate simultaneously to co-evaporate the interface between SiO_2_ and Al_2_O_3_.


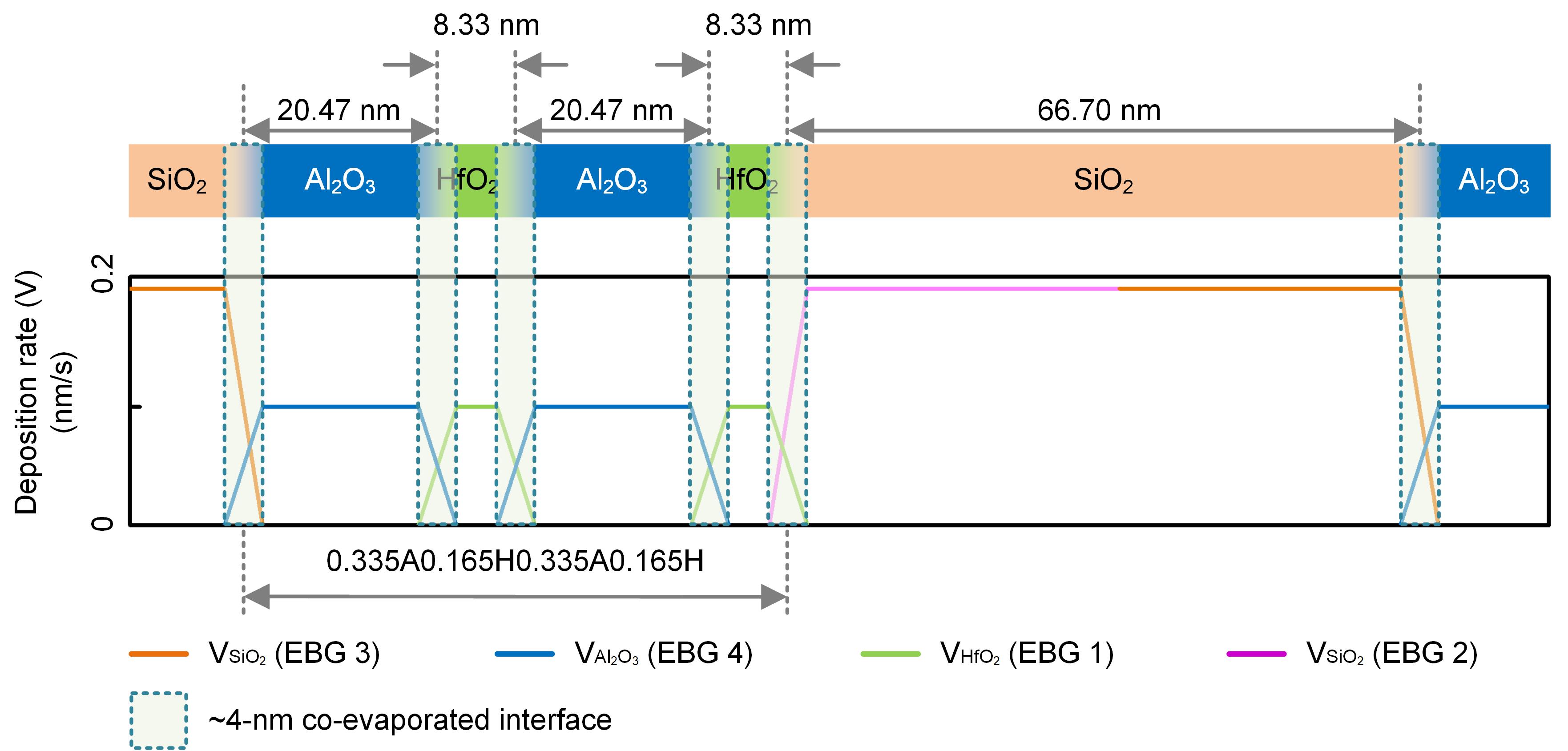


**Figure S2.** The deposition rates of different materials and the EBGs used

The cross-section morphologies of the TCD and NLD coatings characterized by TEM are shown in Figure S3.


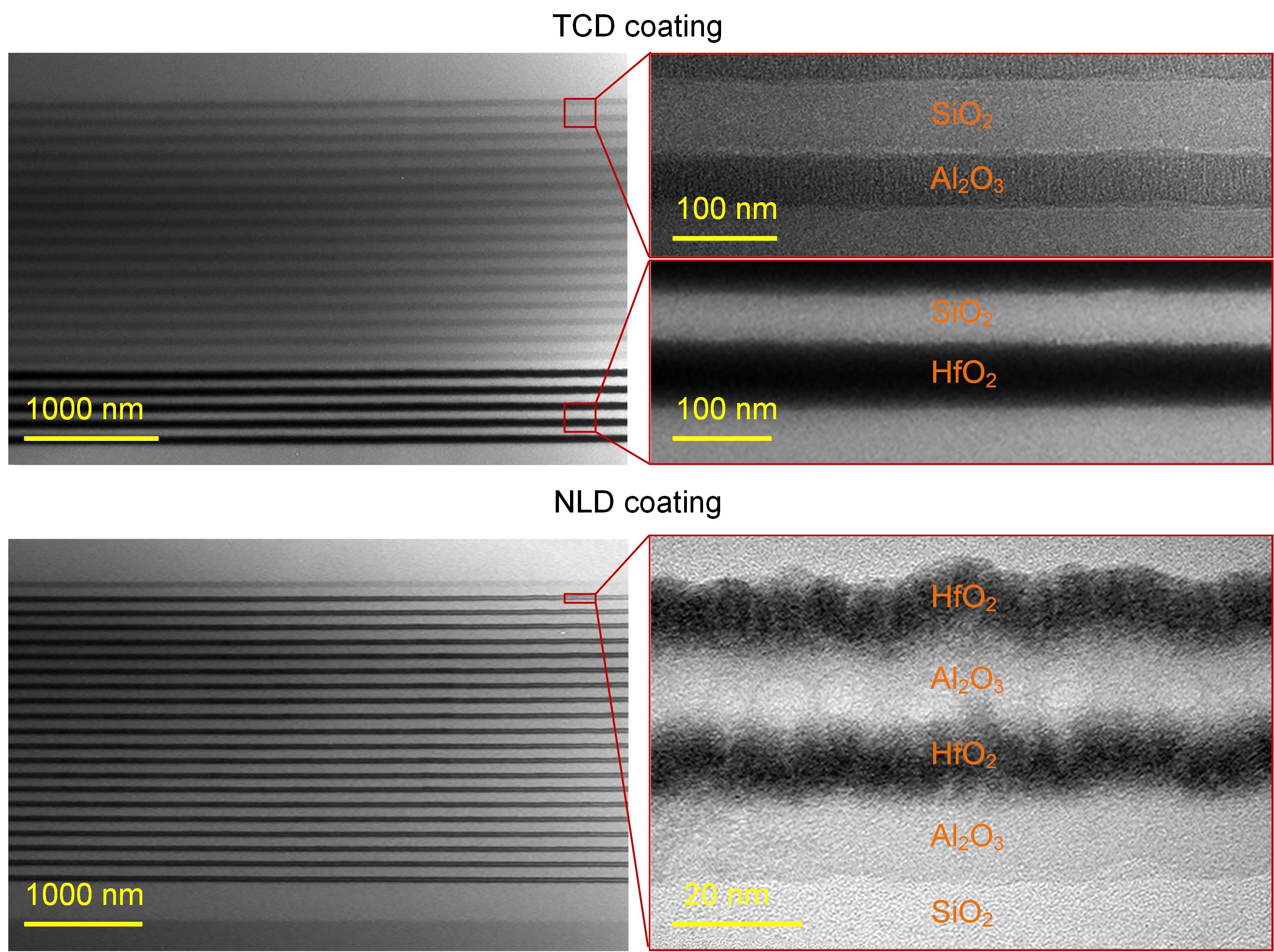


**Figure S3.** The cross-section morphologies of the TCD and NLD coatings

Electron diffraction patterns in selected area of the TCD and NLD coatings are shown in Figure S4. For both TCD and NLD coatings, the selected area electron diffraction pattern shows the crystalline diffraction spots and/or rings.


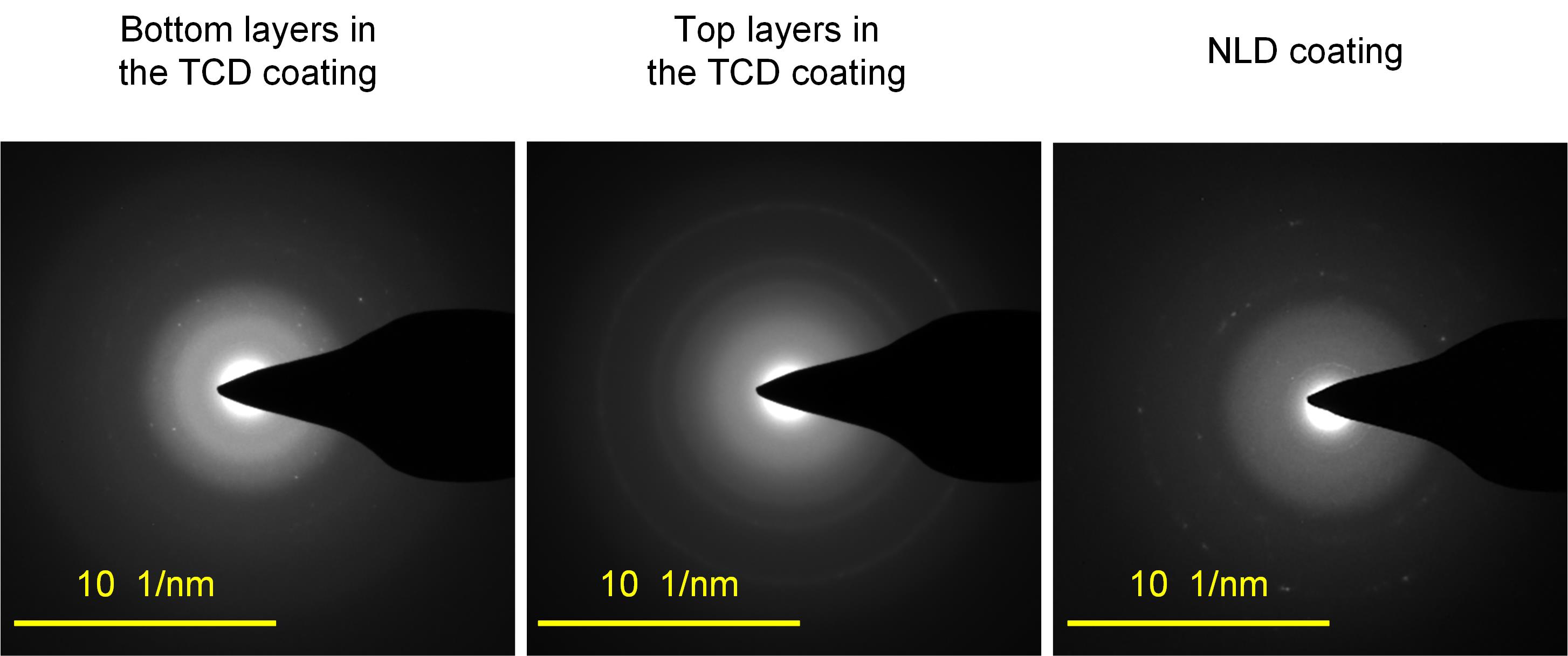


**Figure S4.** Electron diffraction patterns of the TCD and NLD coatings

FEM simulation is used to investigate the E-filed intensification in the TCD and NLD coating caused by nodular defects, a cross-sectional image of nodular defect in the multilayer coating is used as input for simulation. Figure S5 shows the *s* polarized E-field intensity distributions at 355 nm for an angle of incidence of 45° in the multilayer coatings without and with nodular defects.


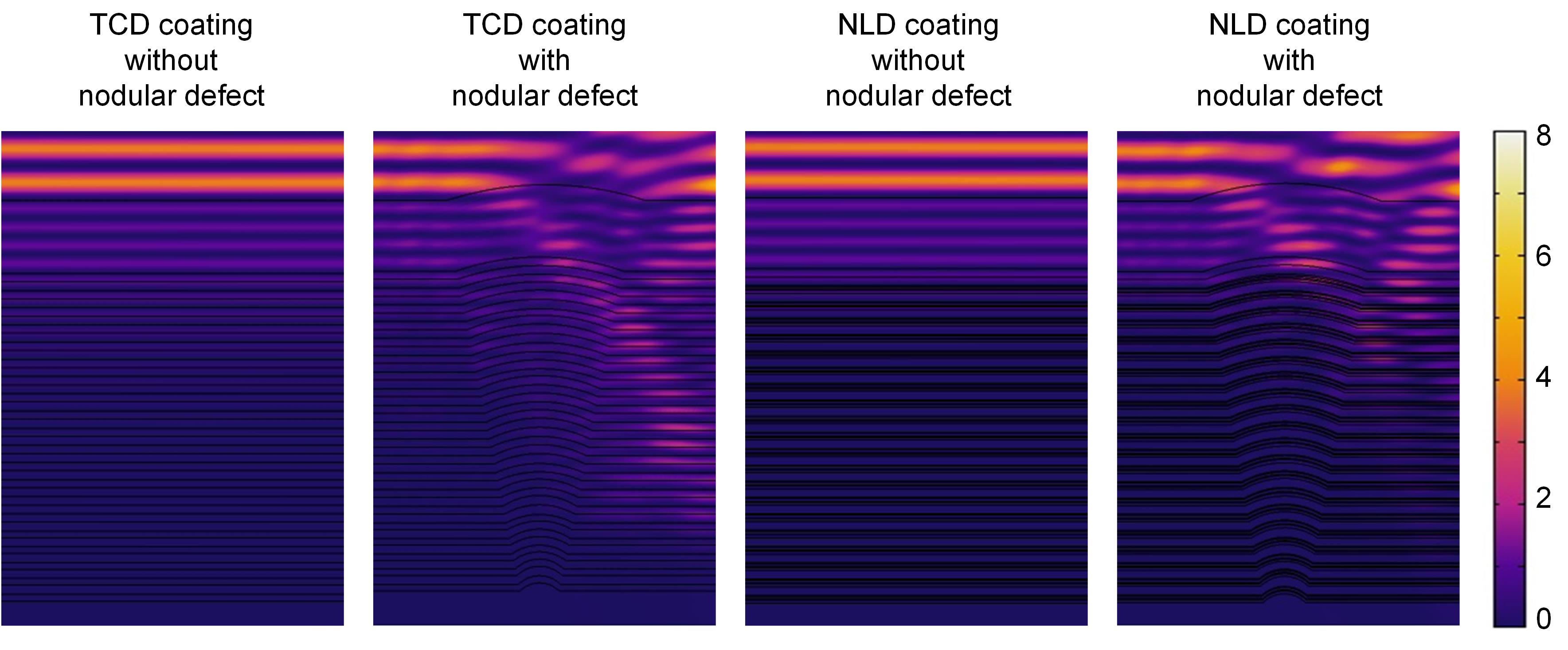


**Figure S5.** E-field intensity distribution in the TCD and NLD coatings
